# Relationships between Gene Expression and Brain Wiring in the Adult Rodent Brain

**DOI:** 10.1371/journal.pcbi.1001049

**Published:** 2011-01-06

**Authors:** Leon French, Paul Pavlidis

**Affiliations:** 1 Bioinformatics Graduate Program, University of British Columbia, Vancouver, British Columbia, Canada; 2 Centre for High-Throughput Biology, University of British Columbia, Vancouver, British Columbia, Canada; 3 Department of Psychiatry, University of British Columbia, Vancouver, British Columbia, Canada; Indiana University, United States of America

## Abstract

We studied the global relationship between gene expression and neuroanatomical connectivity in the adult rodent brain. We utilized a large data set of the rat brain “connectome” from the Brain Architecture Management System (942 brain regions and over 5000 connections) and used statistical approaches to relate the data to the gene expression signatures of 17,530 genes in 142 anatomical regions from the Allen Brain Atlas. Our analysis shows that adult gene expression signatures have a statistically significant relationship to connectivity. In particular, brain regions that have similar expression profiles tend to have similar connectivity profiles, and this effect is not entirely attributable to spatial correlations. In addition, brain regions which are connected have more similar expression patterns. Using a simple optimization approach, we identified a set of genes most correlated with neuroanatomical connectivity, and find that this set is enriched for genes involved in neuronal development and axon guidance. A number of the genes have been implicated in neurodevelopmental disorders such as autistic spectrum disorder. Our results have the potential to shed light on the role of gene expression patterns in influencing neuronal activity and connectivity, with potential applications to our understanding of brain disorders. Supplementary data are available at http://www.chibi.ubc.ca/ABAMS.

## Author Summary

We tested the idea that the “wiring diagram” of the adult brain has a relationship with where genes are expressed. We were inspired by similar work carried out by groups examining the nematode worm *Caenorhabditis elegans*. By using large-scale databases of brain connectivity and gene expression in rodents, we found that many genes involved in the development of the brain show correlations with anatomical connectivity patterns. Some of the genes we found have been implicated in disorders such as autism, which is suspected to affect brain wiring. While the biological causes of the patterns we found are not yet known, we believe they provide new insight into the patterns of gene expression in the brain and will spur further study of this problem.

## Introduction

While the brain can be studied at many different scales and with many modalities, one of the most established is the study of brain regions and their connectivity. These “macroconnections” between neuroanatomically-defined brain regions are thought to number between 25,000–100,000 in the mammalian brain [1], forming a complex network. Knowledge of the “connectome” is used to diagnose neurological disorders such as ischemic stroke, to interpret brain imaging results and to computationally model the brain. There is also growing evidence of connectivity abnormalities in disorders such as autism and schizophrenia [2], [3], [4]. Because of the fundamental importance of the wiring of the brain, there has been a recent push to create more comprehensive “connectome” maps [5], [6], paralleling efforts to understand the brain at the level of genes.

The most comprehensive studies of connectivity have been done in the worm *Caenorhabditis elegans* (at the level of single neurons) and the macaque monkey [7], [8]. Recent work has begun plumbing the properties of these networks, examining node degree distribution [9], network motifs [10], and modularity [11]. It has been shown that anatomical neighbours tend to be connected [12], and there is evidence that wiring cost partially explains network structure [13], [14]. There is also increasing interest in the integration of neuronal connectivity and information about genes. This is in part driven by the fact that many genes show spatially-restricted or varying expression in the nervous system, but in many cases the reasons for the expression patterns are not clear [15], [16], [17], [18].

The idea that gene expression is related to connectivity is not new. For example, the expression of a transmitter must be coupled with expression of appropriate receptors in the postsynaptic target. To regulate neurite outgrowth and plasticity hetero- and homophilic cell adhesion molecules require appropriate expression patterns in connected neurons [19], [20]. In a study of the mouse hippocampus, Dong et al. [21] identified seven genes which are differentially expressed between the dorsal and ventral CA1 field and have a correlated expression pattern in the corresponding projection fields in the lateral septal nucleus. The availability of detailed information on expression patterns in the mouse brain [15], [16], [17] suggests that a global examination of gene expression and connectivity in the mammalian brain would provide additional insights.

While there is no large-scale analysis of gene expression and connectivity in the mammalian brain, three groups have examined this issue in the nematode worm *Caenorhabditis elegans*. The groups used cellular level expression data for a few hundred genes and a neuron level connectivity map [8]. By combining the data, Kaufman et al. [22] used classification and Mantel tests to predict genes involved in synaptogenesis and axon guidance. They concluded that expression profiles of neurons “carry significant information about their connectivity”. Varadan et al. [23] used a different methodology to discover biologically meaningful gene sets that provide connectivity information. Within the resulting gene sets they found high levels of multivariate synergy, suggesting interacting genes are more important than single genes. In a third study, Baruch et al. [24] predicted a neuron's postsynaptic partners using expression patterns of a small number of interacting genes.

In this paper we examine gene expression patterns and macroconnectivity in the adult rodent brain, using data from the Allen Brain Atlas [17] and the Brain Architecture Management System [25], [26]. Our results suggest that in the mammalian brain, as in *Caenorhabditis elegans*, there is a correlation between gene expression and connectivity, and the relevant genes are enriched for involvement in neuronal development and axon guidance.

## Results

**Figure 1:**
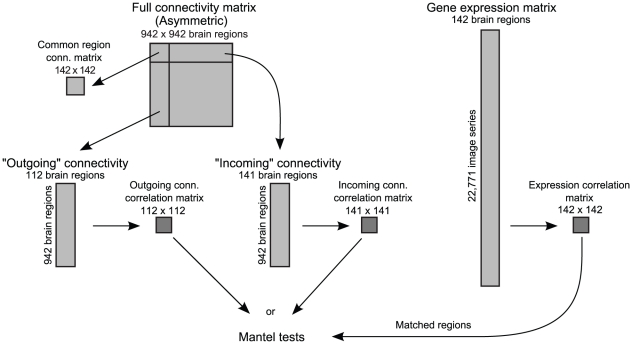
Datasets and correlation matrices used in this paper. Matrices are shown schematically as shaded boxes; arrows indicate steps in the workflow. For example, from the full connectivity matrix we extracted submatrices of “outgoing” or “incoming” connectivity, and compared their correlation matrices with the correlation matrix of the brain region expression patterns.

We obtained data sets of macroconnectivity in the rat brain and gene expression data on mouse (see Materials and Methods and Figure 1). By carefully mapping brain regions across them, we identified 142 distinct (non-overlapping) brain regions in common (the “common” regions; see Materials and Methods). In total these regions account for nearly half of the volume of the brain. A notable omission is many regions of the neocortex, which is not sub-parcellated in our data set.

The expression data set, which is filtered to remove unexpressed genes (see Materials and Methods) consists of the expression levels of 17,530 genes in the 142 regions. Because many genes were assayed more than once in the Allen Atlas (independent “image series” in their terminology), there are 22,771 rows in the expression data matrix. The connectivity data consists of the connectivity profiles of 942 regions with the 142 common regions (Figure 1). In this binary matrix, a value of 1 at index (*i*,*j*) indicates a connection exists between region *i* and region *j*. In most of our analyses, we considered the directionality of connectivity. Of the 142 common regions, 112 have efferent (outgoing) connections, and 141 have afferent (incoming) connections; there are 5216 outgoing connections and 6110 incoming connections. Our results are based on various direct and indirect comparisons of the connectivity and expression data matrices or their corresponding correlation matrices.

We began our study with some relatively simple analyses designed to explore the relationship between connectivity, gene expression and other parameters such as spatial distribution and size of brain regions.

We first tested the simple hypothesis that regions which are connected might have more similar expression patterns. This is in effect a more global search for patterns like the ones identified by Dong et al. [21] (note that the CA1 subregions studied by Dong et al. were not represented in our data). To do this we compared the distribution of correlations in expression profiles for regions which are connected to the distribution for regions that are not connected (Figure S1). We found that on average, regions that are connected (ignoring directionality; 912 connected pairs among the 142 regions) have more similar expression profiles than the 8,187 non-connected region pairs (0.79±0.06 for connected; 0.76±0.06 for unconnected; p-value<2.2×10^−16^, t-test). This is an initial indication that structural connectivity and gene expression are related.

We then examined the degree of connectivity of a region with its expression profile. The degree of connectivity is computed by summing the columns of the connectivity matrix in Figure 1. The correlation of this vector was then computed with each gene expression profile (the rows of the expression matrix). After correcting for multiple testing, 887 and 1127 genes (represented by 929 and 1175 Allen Brain Atlas image series, respectively) had expression levels positively and negatively correlated with the number of connections, respectively. The highest rank correlations between expression levels and connectivity degree were ∼±0.64. While the interpretation of this result is not clear (a Gene Ontology annotation enrichment analysis did not yield any strong patterns), we noted that all three neurofilament cytoskeleton genes (light [NCBI gene ID:18039], medium and heavy neurofilament polypeptides, Nefl-3) are negatively correlated with connectivity; that is, they are expressed at higher levels in regions that have few connections. Neurofilament content is correlated with axonal diameter and length, with enrichment in motor and long-projecting neurons [27], [28], [29] and our results suggest another relationship with connectivity.

We found that the size of a region is significantly correlated with its connection degree (Spearman's rank correlation, ρ = 0.22). We also noted that the more posterior the region, the fewer connections it has (ρ = 0.55). Regions containing motor neurons that project long axons to the spinal cord or muscles were found to have significantly fewer connections (they also tend to be in posterior locations; p-value = 1.32×10^−6^, Wilcoxon–Mann–Whitney test). Table S1 provides brain region statistics for degree, location and motor classification.

While the above analyses suggest some interesting generic patterns relating connectivity to expression and other parameters, they are not able to expose more complex relationships. Like Kauffman et al. [22] and Varadan et al. [23], we hypothesized that expression patterns carry information about specific neural connectivity patterns involving multiple regions. To test the global correlation between expression and connectivity profiles we used the Mantel test. Unlike the test used above to examine the relationship between pair-wise connectivity and expression patterns (using the direct connectivity matrix), here we are asking if the similarity of the connectivity profiles of two regions is related to the similarity of the expression profiles of the two regions, regardless of whether those two regions are themselves connected. In this analysis we are comparing the correlation matrices for the expression data set and the connectivity data (Figure 1).

A key finding is that, as in *Caenorhabditis elegans* (at the level of individual neurons), we find that brain regions that have similar connectivity patterns tend to have similar patterns of gene expression. The Mantel correlation (“correlation of correlations”) between expression and incoming connectivity patterns (141 regions) is 0.248 (p-value<0.0001). Using the outgoing connectivity profiles for 112 regions yielded a correlation of 0.226 (p-value<0.0001). This relationship holds separately for some of the 5 major neuroanatomical divisions in the Allen reference atlas. For outgoing profiles the Mantel test is significant at p-value<0.001 for the interbrain (r = 0.42), cerebrum (r = 0.30) and hindbrain (r = 0.21) divisions but not midbrain or cerebellar divisions. For incoming connectivity only the cerebrum (r = 0.29) and interbrain (r = 0.34) divisions have significant Mantel correlations with expression. We note that unlike our observation of similar expression profiles among connected regions, here we are comparing connectivity patterns of regions, which does not require that the regions be connected to each other.

**Figure 2:**
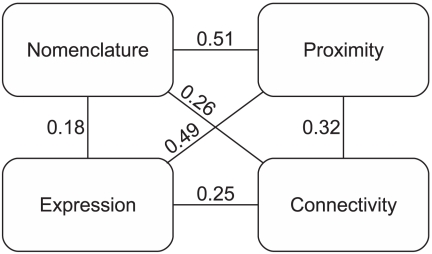
Mantel correlations between different matrices. “Nomenclature” and “Proximity” refer to the two different measures of spatial distance that we used (see Materials and Methods). The 141 regions with incoming connectivity information were used to generate the correlations for this figure.

One factor in this analysis is that regions which are near each other tend to be connected [12] and also might be expected to have higher correlations in expression patterns (because nearby regions will tend to be of the same embryonic origin, for example). This will tend to obscure the degree to which expression is specifically correlated with connectivity (and in turn obscure the degree to which expression is specifically correlated with location). We assessed the overall degree of spatial autocorrelation by performing the Mantel test as above, but comparing expression or connectivity to a matrix representing physical distance or, alternatively, nomenclature distance (relationships in the nested hierarchy of brain regions). As expected, the Mantel test results are all significant (Figure 2). The connection data (r = 0.32; p-value<0.001, Mantel test) appears to be less spatially autocorrelated than expression (r = 0.49; p-value<0.001, Mantel test).

**Figure 3:**
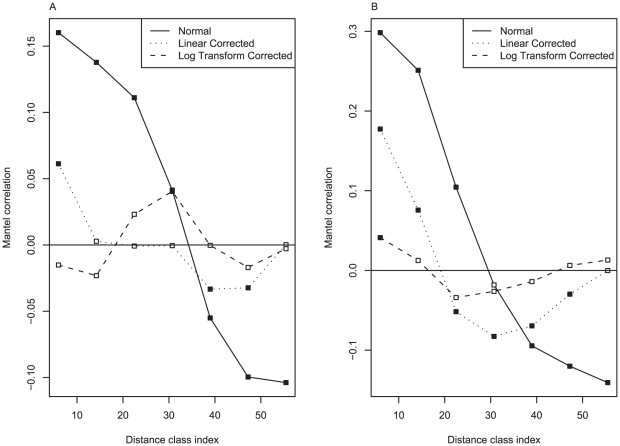
Connectivity (A) and expression (B) Mantel correlograms for uncorrected, linear and log transform corrected spatial distance matrices. Filled squares mark distance classes with significant spatial correlation after multiple test correction.

We visualized the spatial correlation structure with Mantel correlograms (Figure 3). The Mantel correlogram displays the correlation between a data matrix and a matrix formed by grouping region pairs into distance classes. The correlogram will not be flat if it is possible to predict the distance class of a pair based on connectivity or expression correlations alone. As shown in Figure 3, there is indeed an effect of distance on the correlation between connectivity and expression. We therefore attempted to correct our analysis for the effect of spatial autocorrelation, using regression. We calculated regressions between the distance and expression or connectivity correlations for all region pairs. The residuals of these regressions provide proximity-controlled correlations. As shown in Figure 3, an improvement in the correction is obtained when using log-transformed distances.

Using the log-transformed distance matrix from above, we can control for spatial autocorrelations by applying the partial Mantel test [30],[31]. The partial Mantel test applies the same regression mentioned above to both the connectivity and expression similarity matrices. Then a standard Mantel test is calculated between the two spatially-corrected residual matrices. We found that after correction, the partial Mantel test between connectivity and expression remains significant, indicating the relationship is not entirely due to neighbourhood effects. However as expected the correlations are lower. Using the spatial correction, the correlation between incoming connectivity and expression is 0.109 (p-value = 0.008, Mantel test), for outgoing it is 0.126 (p-value = 0.001, Mantel test; summarized in Figure S2). As a further confirmation for the effectiveness of the correction based on spatial distance, we found that the correlation between nomenclature distance and expression or connectivity correlation drops substantially, though the correlations are still significant (Mantel correlation −0.089 for expression, p-value = 0.006; 0.11 for connectivity, p-value<0.001). This incomplete correction is perhaps not surprising as the nomenclature hierarchy reflects connectivity as well as spatial location.

**Figure 4:**
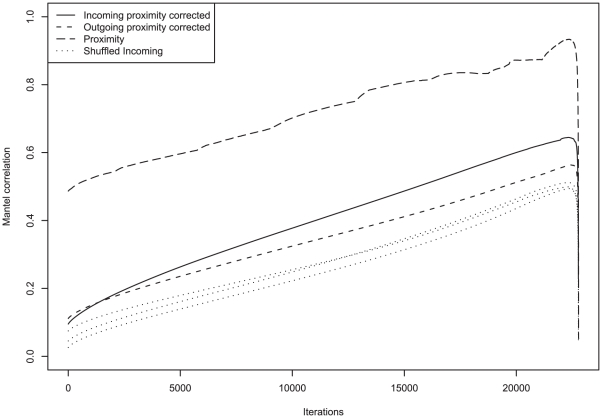
Optimization of Mantel correlation by iteratively removing image series. Each curve documents the correlation across iterations (as genes are greedily removed).

The above tests use expression information for all expressed genes in the Allen Brain Atlas, but we expect that many genes will not contribute any information on connectivity. To find the most informative genes, we applied a greedy algorithm that identifies subsets of the data which maximize the correlation between connectivity and expression patterns (see Materials and Methods). Figure 4 displays the change in the Mantel correlations as genes are iteratively removed. As shown in Table 1, this yields much smaller sets of genes (357 and 433 for outgoing and incoming, respectively) and much higher Mantel correlations (0.56 and 0.65 for outgoing and incoming connectivity respectively). Figure S3 provides a visualization of these results by intersecting region pairings with high expression and connectivity correlations. As a control, we performed the same procedure on multiple shufflings of the expression data, yielding a maximum correlation across ten runs of r = 0.42 and r = 0.51 for outgoing and incoming respectively. We also carried out the same procedure for the spatial correlations instead of connectivity, yielding a “spatial proximity” list of 401 genes and a Mantel correlation of 0.934. Eighty-five image series (89 genes) were found to overlap between the lists for incoming and outgoing connectivity, which is not surprising because there is a fair amount of reciprocal connectivity. Twenty-one image series (31 genes) overlap across the spatial proximity list and one or both of the connectivity gene sets, suggesting that for the most part, different genes provide information about connectivity and proximity. The top twenty image series for the rankings are provided in Table 2 (full results are available as Tables S2, S3, and S4). If we consider just the top 20 genes, the Mantel correlations are 0.516 (incoming), 0.460 (outgoing) and 0.590 (proximity). As an additional control, we found that the correlations obtained for the optimized gene sets are robust to the completeness of the connectivity network (tested by, for example, randomly removing brain regions and recomputing the Mantel correlations). Thus, while the connectivity map of the rodent brain is incomplete, the correlations with expression appear robust.

**Table 1:** Peak correlation and size of optimized Mantel tests.

Name	Peak Correlation	Size (image series)
Incoming	0.645	452
Outgoing	0.564	374
Proximity	0.934	420

**Table 2:** Top twenty genes for proximity and proximity-controlled incoming and outgoing Mantel tests.

Incoming			Outgoing			Proximity		
Rank	Symbol	Imageset	Rank	Symbol	Imageset	Rank	Name	Imageset
1	Nrp2	80514091	1	Pgrmc1	797	1	Nup37	68795447
1	D4st1	74657927	1	Slc25a37	68445000	1	Klrg1	69735903
3	Acadvl	227161	3	Pcp2	77413702	1	Dnahc1	73520818
4	Pgrmc1	797	4	Galr1	80514053	1	Pus1	532760
5	8030411F24Rik	74580853	5	1700054O13Rik	69117086	1	Mm.359340	71209910
6	Gda	70276867	6	Plk1s1	70295882	1	Tm2d3	77414123
7	Mdfi	275690	7	Alpk3	71574473	1	LOC433436	73636096
8	3110082D06Rik	74581400	8	Lrrn6c	72128919	8	Gba2	68844337
9	Lyzs	68191492	9	Gm47	70565879	9	Prrg2	276063
10	Atad2b	71496393	10	Cpne5	544709	10	Ccdc137	1979
11	Slc5a2	68632936	11	Nmbr	77332086	11	Col5a3	74272917
12	Dbnl	74819497	12	Trim52	70205626	12	Kcnk2	75147764
13	Dmp1	74511936	13	AI427122	71495698	13	Comt	68301371
14	Gata3	73931427	14	Slc44a4	68321886	14	Bcl2l12	71064289
15	Rgs9	73521819	15	Nrp2	80514091	15	Mtif2	68341663
16	En2	69288944	16	Anxa3	69526665	16	Eomes	80516770
17	Wisp2	68523207	17	A930033C23Rik*	74300717	17	Gcnt1	68546476
18	Cypt3	80474702	18	Tac2	77279001	18	LOC433088	70722898
19	F2rl1	199391	19	C1qtnf9	70228041	19	Mrpl45	70919854
20	1700018L24Rik	74634791	20	Kirrel1	71613657	20	Gda	70276867

We next examined the expression patterns of the optimized gene lists in more detail. It was of interest to determine, for example, if all the genes had similar expression patterns, which would suggest a single overwhelming signal in the data. A hierarchical clustering and visualization of the expression patterns of the optimized gene sets suggested that the patterns are in fact diverse (Figures S4 and S5). This is supported by a comparison of the distributions of gene-gene correlations within the optimized outgoing list, which are on average slightly lower than the full data set (0.10±0.21 for top outgoing genes; 0.15±0.21 for all genes; p-value<2.2×10^−16^, t-test, Figure S6). This suggests that many different gene expression patterns are contributing to the overall correlation between connectivity and gene expression.

**Figure 5:**
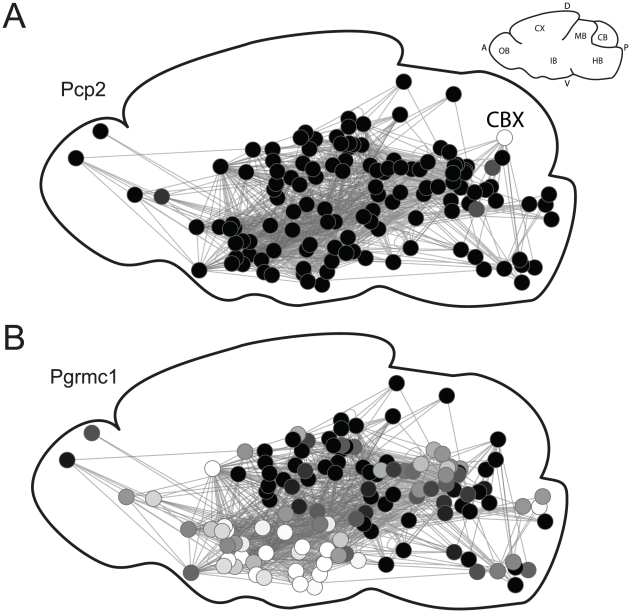
Connectivity in the context of Pcp2 (A) and Pgrmc1 (B) expression. The connectivity map is a 2-D projection of the network on the saggital plane. Each node represents a brain region (placed at the center of the region as measured in the Allen reference atlas). Expression levels are depicted as shades of grey, with lighter shades indicating higher expression. Pcp2 expression is restricted to the cerebellar cortex (CBX), while Pgrmc1 tends to be expressed highly in both regions of connected pairs. The small inset brain diagram provides orientation (anterior (A), dorsal (D), ventral (V) and posterior (P)) and the locations of the olfactory bulb (OB), cortex (CX), interbrain (IB), midbrain (MB), hindbrain (HB) and cerebellum (CB).

Figure 5 shows the expression patterns for two genes that rank high in the “outgoing” gene list, overlaid on schematics of the connectivity data. In Figure 5A, we show the pattern for Pcp2 (Purkinje cell protein 2; Figure 5A). Although Pcp2's function is unknown, it is almost exclusively expressed in the projection neurons of the cerebellar cortex (Purkinje cells). We did not expect this specific expression pattern to carry information about connectivity because no other regions express Pcp2. However, the connections of the cerebellar cortex are also unique and specific: of the 112 outgoing regions, 69 place the cerebellar cortex in the bottom tenth percentile of similar regions based on proximity controlled connectivity. As a result, the optimization procedure finds that Pcp2's expression pattern marks the cerebellar cortex's unique connectivity profile. Figure 5B shows the expression pattern of Pgrmc1 (Progesterone membrane component 1), a gene that may play roles in axon guidance [32], [33]. In contrast to Pcp2, which is expressed in only one brain region, expression of Pgrmc1 in two regions is correlated with a connection between them (Figure S7). Thus, clusters of highly connected regions tend to show higher levels of Pgrmc1 expression (Figure 5B). While the strong relationships shown in Figure 5 are not representative of the data set as whole, they serve to illustrate how expression patterns can contain information on connectivity.

One concern about using high-throughput *in situ* hybridization data might be the potential for artifacts. While all of the image series we used had passed the Allen Brain Atlas project's (ABA) own quality control criteria, we did note occasional spatial artifacts such as dust or bubbles, though there was no indication such problems were more common in the genes we ranked highly. In addition, while there is good evidence that the ABA data are reliable, with a high quantitative and qualitative agreement with other data [34], [35], there are genes (∼6% in ABA) for which ABA has disparities [35] and a few of those genes show up in our results (at approximately the expected proportion; see Dataset S1). To help address these concerns, we extracted a higher-confidence subset of results by considering genes measured more than once in the Allen Brain Atlas. These “duplicate” image series vary primarily by the RNA probe sequence used and the plane of section (sagittal vs. coronal), and it seems unlikely that results which are concordant across image series would be due to expression analysis artifacts. Seventeen genes in our top outgoing connectivity list have two concordant image series. In the case of incoming connectivity, 16 of the genes on our list are represented by at least two image series (Rprm has three, and Calb2 has four of its 20 image series across the atlas). We refer to these as the “high-confidence” lists.

The next stage of our analysis was to consider in greater detail the types of genes which are correlated with connectivity. We accomplished this through a combination of Gene Ontology (GO) annotation enrichment analysis and manual review of the literature relating to the genes, particularly those on our high-confidence lists. We specifically hypothesized that genes that play roles in neural development might be found, as suggested by previous work on *Caenorhabditis elegans* [22], [23].

In agreement with this hypothesis, our Gene Ontology analysis of the “outgoing” list revealed significant enrichment in categories related to neuronal development (Table 3; note that many of the top groups have overlapping gene members. No GO terms were significant for the “incoming” or “proximity” lists. Full GO analysis results are in Table S5). A manual examination of the connectivity top gene lists (Tables S2 and S3) makes it clear that this is due to the presence of many different genes that play a variety of roles in neuronal development, but axon guidance was a prominent theme. Our lists contain a total of 14 members of three major axon guidance families (Semaphorin, Ephrin, and Slit families) [36] (Table 4). These gene families express cell-surface or secreted proteins that function to provide guidance signals to growing axons. This was most striking for the Semaphorin family, with ligands, receptors and co-receptors appearing in the incoming or outgoing top gene lists (Table 4). Six of the 17 genes from the high-confidence “outgoing” list function in neuronal development and axon guidance. Two of these six, Gpc3 and Hs6st2 encode a heparan sulfate proteoglycan and a heparan sulfate sulfotransferase respectively. Two additional heparan sulfotransferases, Hs3st1 and Hs6st1 appear with one image series on outgoing top gene list. Heparan sulfate proteoglycans are membrane proteins that have been linked to neurogenesis, axon guidance and synaptogenesis [37]. Hs6st2 has been specifically linked to retinal axon targeting in Xenopus [38]. Another gene on the high-confidence list is the L1 cell adhesion molecule (L1cam), a recognition molecule involved in neuron migration and differentiation [39]. Vesicle-associated membrane-protein (Vamp2) is another gene connected to connectivity through two image series; in addition Vamp1 occurs once in the outgoing list. Recently Vamp2 has been linked to attractive axon guidance but not repulsion in chick growth cones [40]. Neurturin is another high-ranking gene with two image sets linked to outgoing and one linked to incoming. Neurturin is well known to promote neuronal survival and induce neurite outgrowth [41]. Lastly, Serinc5 is enriched in white matter and Inuzuka et al. [42] suggest its major role is to provide serine molecules for myelin sheath formation.

**Table 3:** Top twenty GO groups enriched in the proximity controlled outgoing ranked gene list.

Name	ID	Group Size	Hits	P-value	Corrected P-value
neuron projection development	GO:0031175	186	16	0.00000	4.63E-003
cell morphogenesis involved in differentiation	GO:0000904	183	13	0.00013	0.05
cell projection morphogenesis	GO:0048858	157	12	0.00012	0.06
cell part morphogenesis	GO:0032990	166	12	0.00020	0.06
cell migration	GO:0016477	189	13	0.00018	0.06
axonogenesis	GO:0007409	145	12	0.00005	0.07
cell morphogenesis involved in neuron differentiation	GO:0048667	157	12	0.00012	0.07
neuron projection morphogenesis	GO:0048812	154	12	0.00010	0.08
positive regulation of secretion	GO:0051047	27	4	0.00227	0.45
negative regulation of cell communication	GO:0010648	150	9	0.00438	0.45
heparan sulfate proteoglycan biosynthetic process	GO:0015012	5	2	0.00420	0.45
lymphocyte differentiation	GO:0030098	83	7	0.00177	0.47
leukocyte activation	GO:0045321	161	9	0.00691	0.47
B cell differentiation	GO:0030183	33	4	0.00480	0.48
positive regulation of cell-cell adhesion	GO:0022409	5	2	0.00420	0.48
negative regulation of neuron differentiation	GO:0045665	28	4	0.00260	0.48
regulation of neuron differentiation	GO:0045664	82	6	0.00746	0.48
epithelial cell development	GO:0002064	19	3	0.00689	0.48
central nervous system neuron axonogenesis	GO:0021955	13	3	0.00223	0.48
lymphocyte activation	GO:0046649	140	8	0.00936	0.48

**Table 4:** Members of three canonical axon guidance families appearing in our connectivity and proximity top genes lists.

Name	Connectivity	Proximity
**Semaphorins and receptors**	Sema3a, Sema6a, Nrp1, Nrp2, Plxna2, Plxnb2	Sema3a
**Ephrin/Eph**	Ephb1, Epha7, Epha8	Efna1, Epha7
**Slit/Robo**	Slit1	Slitrk4

In the case of genes correlated with patterns of incoming connectivity, 4 of the 16 of the genes on our high confidence list have previously suggested roles in brain connectivity. Neurensin-1 shows up with two image series and is known to be involved in neurite extension [43]. Recently, Stat5a has been labelled a key effector molecule in the mammalian CNS, affecting axon guidance in the spinal cord and cortex [44]. Thirdly, Uchl1 is mutated in the GAD mouse strain that presents axon targeting and genesis defects [45]. Finally, ciliary neurotrophic factor receptor (Cntfr) appears twice on the top ranked list and is known to promote neuron survival and plays important roles in nervous system regeneration and development [46], [47].

Another trend we notice from the GO results is that groups of genes with negative regulatory roles are much more prominent than the corresponding “positive” groups (e.g., “negative regulation of neurogenesis”) though these groups are not statistically significant after multiple test correction. The high ranking of these terms (which share members) is due to 11 genes: Hdac5, Notch3, Nrp1, Cd24a, Cit, Apc, Nr2e1, Ptk2, Gpc3, and Runx2. The “negative” aspect of the function of these genes varies but all have roles in neuronal development and/or plasticity. For example Nrp1 is a coreceptor for semaphorins and triggers inhibition of axonal growth [48], while Hdac5 is a histone deacetylase whose activity is associated with repressed chromatin conformations that are altered after addictive stimuli [49].

We also conducted a search among our high-confidence list for genes whose homologs are implicated in human disorders of the nervous system. We found evidence for such a role for five of the 30 genes. Prominent among the five is L1Cam, defects in which cause several brain disorders including partial agenesis of the corpus callosum [50]. Two genes in the high confidence lists have been linked to heritable forms of Parkinson's disease (alpha-synuclein (Snca) [51] and Uchl1 [52]). Finally, two genes have been linked to autistic spectrum disorder (ASD). The human homolog of Cadps2 has been linked to autism and lies in the 7q autism susceptibility locus (AUTS1) [53], [54]. Another, Btg3 is in a genetic locus linked to autistic children characterized by a history of developmental regression [55]. By examining our expanded list of genes, we found several more of our connectivity linked genes are in AUTS1 and have been studied in the context of autism: Reln [56], Mest [57], Ptprz1 [58], Dpp6 [59] and En2 [60]. To further explore the potential connection between our results and autism, we downloaded all autism candidate genes from the AutDB database [61]. Of those genes, 163 were available in our dataset, and 17 appear in at least one of the connectivity linked lists (14 for incoming connectivity and Nrp2, Cadps2, Ntrk1,and Apc appear in both incoming and outgoing lists). The probability of this occurring by chance is 0.00029 (hypergeometric test; considering the incoming list alone the p-value is 5.43×10^−5^). In contrast, the proximity-ranked list contains only 5 genes in the AutDB set (p-value = 0.32).

## Discussion

Our analysis revealed a number of interesting relationships between gene expression and patterns of connectivity in the adult mammalian brain. Our key finding is that genes whose expression patterns carry information on connectivity are enriched for genes involved in neural development, and axon guidance in particular. While our results are based on analysis of the brains of rodents, it is of potential importance that many of the genes we identify have human homologs implicated in disorders of the nervous system including ASD. Because there is an increasing interest in the idea that ASD and other disorders are in part due to abnormalities in connectivity [4], [62], and given the heritability of many such disorders, the relationship between gene expression and connectivity is pertinent. The enrichment of homologs of autism candidate genes in our results suggests that these patterns could be relevant to the understanding of behavior in autism and potentially avenues for treatment.

To our knowledge ours is the first study comparing gene expression and connectivity in mammals at a global level. Interestingly, a previous focused examination of the correlation between expression and connectivity for two brain regions identified some of the same genes we did. Dong et al. [21] examined correlations between genes that are differentially expressed between the dorsal and ventral hippocampus (which we were not able to treat as separate regions in our analysis). For nine of their genes, they observed matching expression patterns in a connected brain region, the lateral septal nucleus. Three of these seven genes appear on our connectivity correlation lists (Gpc3, Man1a, Wfs1); this is unlikely to occur by chance (p-value = 0.0045, hypergeometric test). In contrast, none of the seven appear on the proximity gene list.

We stress that because what we observe are correlations, it is difficult to ascribe a definite mechanism or meaning to the patterns. In addition, in absolute terms the Mantel test correlations may seem low when we considered all genes. However, we do obtain a correlation of 0.65 between gene expression patterns and proximity-controlled incoming connectivity after gene selection. We also point out that at the neuron to neuron level in *Caenorhabditis elegans*, Kaufman et al. [22] reported statistically significant correlations of 0.075 and 0.176 between expression and incoming and outgoing connectivity, respectively. Thus the patterns we observe in the adult mammalian brain are at least as strong as those observed in previous studies. An obvious question is whether the signals we observe are strong enough to predict patterns of connectivity. Unfortunately, while the signals we observe are statistically significant, they are not strong enough to allow prediction of connections based on expression patterns. Kaufman et al. [22] attempted this with their data and achieved very low accuracy. Using similar data, Baruch et al. [24] attained statistically significant results in predicting the direction of connectivity between neurons known to be connected or which share a common synaptic partner. Using advanced imaging techniques on human subjects, Honey et al. [63] attempted to predict diffusion tensor imaging (DTI) based cortical connectivity from fMRI functional connectivity. By setting thresholds on functional connectivity, they achieved an AUC value of 0.79 that could predict only ∼6% of inferred DTI connections [63]. Despite these limitations, our results suggest some underlying models that in turn provide some testable hypotheses.

Many of the genes we find to be associated with connectivity patterns in the adult are thought to be primarily active in the developing brain, when large-scale connectivity is determined. The reasons for expression of these genes in the adult brain is not fully understood, though there is evidence in some cases that they continue to play roles in the maintenance or tuning of neuronal connectivity at finer scales [16], [64]. There is even less known about why the genes show regionally restricted patterns in the adult brain. Our results are the first to link the expression signatures of some of these genes to macroscopic connectivity. Our results have at least two possible biological interpretations. One is that the expression patterns in adulthood are a “residue” of the developmental pattern that reflects processes occurring when connectivity is laid down, but that the adult expression pattern is not causally related to connectivity at the scale we studied. An alternative is that the expression patterns in adulthood are functionally relevant with respect to connectivity, perhaps in modulating activity in certain pathways. The patterns we identified could be used to design experiments to distinguish between these alternatives.

While we have provided evidence for a relationship between connectivity and gene expression in the mammalian brain, our analysis is surely hindered by the incompleteness of connectivity and expression information. There are many brain regions for which we had expression data but no connectivity. While some of these regions might never have been studied, there are many reports in the literature that are not included in the current connectivity databases. Advances in the generation of connectivity information from new experiments or from more complete use of existing reports will be essential. The availability of additional expression data would also improve our ability to interpret the patterns we observe. In particular, having detailed information on gene expression patterns during development, and their relationships to the developing projection patterns in the brain, could permit stronger inference of causal relationships. A final limitation is that the structural connections we use cannot be easily linked to specific states or functions of the brain. Because of this we could only interpret our results in the context of gene function information. It would be of interest to employ functional connectivity data to link gene expression to more dynamic and task specific states of the brain, especially in the context of genetic variation.

## Materials and Methods

### Neuroanatomical Connectivity Data

For neuroanatomical connectivity knowledge, we used the Brain Architecture Management system (BAMS). BAMS contains extensive information about neural circuitry curated from neuroanatomical atlases and tract tracing experiments [25], [65]. The version of the BAMS database we use contains 7,308 structural connections between 961 rat brain regions and is accessible via bulk download (http://brancusi.usc.edu/bkms/xml/swanson-98.xml). Instead of parsing the original XML we used a converted semantic web version created by John Barkley (http://sw.neurocommons.org/2007/kb-sources/bams-from-swanson-98-4-23-07.owl). The BAMS system stores information on projection strength, number of reports, report citations and absence of connections but it is not available in the database version we obtained. However, directions of the neuroanatomical connections are known, allowing splitting of our analysis between incoming and outgoing connection profiles.

The BAMS curators comprehensively studied the bed nuclei of the stria terminalis (BNST) and indicate that its connection matrix is considered complete [65]. We were concerned that this unusually well-studied region would bias our results, as it has more known connections than the other regions (we considered regions that lack a documented connection to be unconnected). For example, it has over seven times the average number of outgoing connections. To reduce this bias in the dataset, we removed connection information for the BNST and its subparts. We do not suspect the quality of these connections but wished to prevent one well-characterized region from being overrepresented. We believe the complete connectivity matrix of the BNST will be very valuable for future focused analysis.

### Gene Expression Data

We considered using gene expression profiles from SAGE and microarray experiments, but spatial resolution was too low. Therefore we used high-resolution colourmetric *in situ* hybridization (ISH) measurements produced by the ABA [17]. The complete expression matrix from the ABA (kindly provided by the Allen Institute for Brain Research) consists of 5,380,137 entries formed by 25,991 ISH image series and 207 brain regions. In many cases a gene was assayed more than once, using a different probe or plane of sectioning. The ABA provides values for expression “energy”, “level” and “density” across a region. Because level and density had a large fraction of data missing (∼40%) we choose to use expression energy (3% missing). Expression energy is defined as the sum of expressing pixel intensities normalized by the number of pixels in a region. The natural logarithm of expression energy values formed our gene expression matrix. Genes that do not have detectable expression in the ABA were removed. The list of non-expressing genes list was provided in Lein et al. as supplementary data [17]. After removing the non-expressing genes the final gene expression profiles contain 22,771 image series representing 17,530 genes.

### Neuroanatomical Matching and Selecting

The names of brain regions are formalized in hierarchies both in BAMS [26], [66] and the ABA data [67], but the schemes are not identical. In addition, the BAMS dataset contains information at a finer neuroanatomical resolution than ABA. To maximize the use of connectivity information, we created connection profiles of coarser scale by using an up-propagation procedure. Up-propagation maps the brain region to its parent region until the desired level in the neuroanatomical hierarchy is reached. This procedure was applied to all connection pairs in BAMS. For example, a connection between region A and region B will be expanded to the set of all possible connections between the neuroanatomical parents of both region A and region B. To prevent enrichment of up-propagated connections we kept regions that had zero connections to the ABA mapped regions.

Although the two datasets have common objects - brain regions, the organisms differ. The rat brain with a wealth of neuroanatomical information is bigger and for some regions like the cerebellum, more complex. In contrast, genetics and molecular research is more commonly performed on the smaller mouse brain. For this work we considered neuroanatomical differences between the mouse and rat to be minor at the level of granularity we used [68]; for example, the Paxinos mouse atlas was guided by several rat brain atlases [69], and brain regions names largely coincide between the two. These common names allowed quick lexical mapping for most of the regions. To join the two data types we mapped nomenclatures manually. We used primarily a region's name, then secondarily its parent region and spatial borders to pair brain regions. The mappings for the Allen Brain regions are provided in Table S6.

The neuroanatomical atlases from ABA [67] and BAMS [70] provide information on which brain regions are neuroanatomical children or parts of others. These relations create correlations in the gene expression profiles and the connectivity data (due to up-propagation). To negate this effect we used only 149 of 207 Allen brain regions for the primary region list. These remaining regions have no neuroanatomical subparts in the ABA dataset.

The Allen Atlas provides a differing grouping of regions than the BAMS hierarchy. The superior colliculus is one example. The ABA divides its regions into motor and sensory areas, while the BAMS atlas groups the regions into optic, gray and white layers. Differences were resolved by creating “virtual regions” in the BAMS atlas space that contained the corresponding subregions of the Allen Atlas. The connectivity profiles of the mapped regions were joined using a logical OR operation to provide the virtual region's BAMS connections. For example the superior colliculus sensory related virtual region has all of the BAMS connections of the zonal, optic and superficial gray layers. In addition to the superior colliculus, virtual regions were created for the pallidum medial region and nucleus ambiguus.

After mapping of brain regions, the ABA data is an *x* (number of regions in the ABA) by *y* (number of genes) matrix, and the BAMS connectivity data is a square *w* (number of regions in BAMS) by *w* (region) matrix (Figure 1). The two matrices are not directly comparable because the number of regions in BAMS is greater than those in ABA (*w*>*x*). Rather than discarding all information from regions which lack expression information, we use the *x* by *w* submatrix of the BAMS data. Thus each of the *x* regions has a *y*-dimensional expression vector and a *w*-dimensional connectivity vector. This maximizes the use of connection information, but we note that the connectivity profiles include information from regions for which we lack expression information.

### Statistical Tests

Correlations between gene expression values and connection degree were computed using Spearman's rank correlation coefficient (ρ). Connection degree for each brain region is the sum of its propagated incoming and outgoing connections. Significance of the correlation was corrected for multiple testing using the Bonferroni method.

#### Mantel test

To test the hypothesis that there is a statistical relationship between connectivity and gene expression profiles, we apply the Mantel test [71]. The Mantel test is similar to methods previously applied to *Caenorhabditis elegans* data [22]. The Mantel test uses correlation at two levels to measure the relationship between the connectivity and gene expression profiles. First, Pearson correlation for the connectivity and gene expression profiles are computed for each pair of brain regions, resulting in a distance or similarity matrix (Figure 1). The upper triangles of the similarity or distance matrices are then converted to linear vectors. The Pearson correlation of these two vectors is then computed to provide dependence between the connectivity and gene expression profiles for all brain region pairings. The statistical significance is determined from an empirical null distribution. We performed the same analytic procedures used on the ‘real’ data 1,000 or more times using shuffled data. To keep the distribution of the gene expression and connectivity values constant, we shuffle the brain region labels. Significance is determined by counting the number of shuffled datasets that score higher than the non-shuffled result. Mantel correlograms were created using the “mantel.correlog” R library developed by Pierre Legendre (http://www.bio.umontreal.ca/legendre/).

#### Spatial and nomenclature distance matrices

To create the spatial distance profiles we computed Euclidean distance between a given region's centroid and all others, using the Allen Brain Atlas programming interface (API). Further, we created another measure of brain region proximity using the neuroanatomical part-of hierarchy. Similarity between two regions in the nomenclature profile is simply the number of shared neuroanatomical parents. Using these distance matrices we then performed the Mantel test using the spatial, nomenclature and connectivity profiles. Further we applied the partial Mantel test to determine if the correlation between connectivity and expression is still significant after controlling for these proximity measures [30], [31]. Akin to performing a partial correlation, the partial Mantel test uses the residuals of a regression fitted to the distance matrix.

### Gene Ranking and Enrichment

We generate a ranked list of genes so that a gene's rank is proportional to its contribution to the connectivity correlation score. To achieve this we reduce the number of genes in the expression profiles while maximizing the Mantel test correlation score. Since it is not feasible to compute all possible subsets of the image sets, we approximate an optimal candidate list of genes. Again, we take guidance from Kaufman et al. [22] and use a greedy backward elimination algorithm with the Mantel test. Each iteration of the algorithm involves ranking each gene by its contribution to the global correlation, removing the least informative gene, and repeating the test on the remainder. For the connectivity gene rankings we optimized a partial Mantel correlation that modelled proximity in the connection matrix but not the expression correlations (due to computational constraints).

For functional enrichment analysis we employed the ErmineJ software to explore the roles of the candidate genes [72]. Overrepresentation analysis was used on the set of genes removed after correlation reached a maximum. To increase resolution of the genes, NCBI identifiers were used instead of gene symbols. Gene Ontology (GO) groups included in the analysis required 5 to 200 measured gene members and were limited to the biological process division. Benjamini-Hochberg false discovery rate was used to control for testing multiple GO groups [73]. GO groups were sorted by corrected p-value to determine rankings.

For creation of Figures S4 and S5 we employed average linkage hierarchical clustering on both the image series and brain regions. The clustered data was converted to a heatmap using matrix2png with rows normalized to zero mean and variance of 1 [74]. Values were then constrained to the range of −3 to 3.

## Supporting Information

Dataset S1 Gene lists mentioned in the paper. More information about these sets is provided on the supplement website.
(0.07 MB TDS)

Figure S1 Density plot of expression correlation between region pairs.
(0.02 MB EPS)

Figure S2 Mantel correlation between different matrices after controlling for proximity. The 141 regions with incoming connectivity information were used to generate the correlations.
(0.06 MB TIF)

Figure S3 Intersection of the top 10th percentile of brain region pairings for connectivity and gene expression correlations. Using outgoing proximity controlled connectivity. With expression correlation derived from only the top outgoing genes. Colors represent five major brain divisions: cerebellum (yellow), cerebrum (green), hindbrain (blue), interbrain (purple) and midbrain (red).
(0.49 MB TIF)

Figure S4 Heatmap produced by average linkage hierarchical clustering of the top outgoing gene list. Rows are normalized to show expression ranging from low (blue) to high (yellow) with grey representing missing values.
(3.38 MB TIF)

Figure S5 Gene(row) dendrogram from the hierarchical clustering used in Figure S4. Some of the genes mentioned in the text are highlighted in red, showing dispersed clustering.
(0.14 MB EPS)

Figure S6 Density plot of gene-to-gene correlations. Gene to gene correlations were computed within the “outgoing” gene list and all genes.
(0.02 MB EPS)

Figure S7 Pgrmc1 expression levels versus connectivity. For each region pair this plot shows the sum of the two regions' expression in the context of their connectivity.
(0.02 MB EPS)

Table S1 Brain region statistics (appended connectivity).
(0.53 MB XLS)

Table S2 Incoming proximity-controlled top gene set.
(0.20 MB DOC)

Table S3 Outgoing proximity-controlled top gene set.
(0.07 MB XLS)

Table S4 Spatial proximity top gene list.
(0.08 MB XLS)

Table S5 Gene Ontology group analysis results for all three top gene sets.
(1.11 MB XLS)

Table S6 Mapping of Allen Reference Atlas Brain regions to Brain Architecture Management system regions.
(0.03 MB XLS)
