# Supplementary figures and images for: Relationships between Gene Expression and Brain Wiring in the Adult Rodent Brain
Source: PLoS Comput Biol. 2011 Jan 6;7(1):e1001049. doi: 10.1371/journal.pcbi.1001049 (PMC3017102; doi:10.1371/journal.pcbi.1001049)

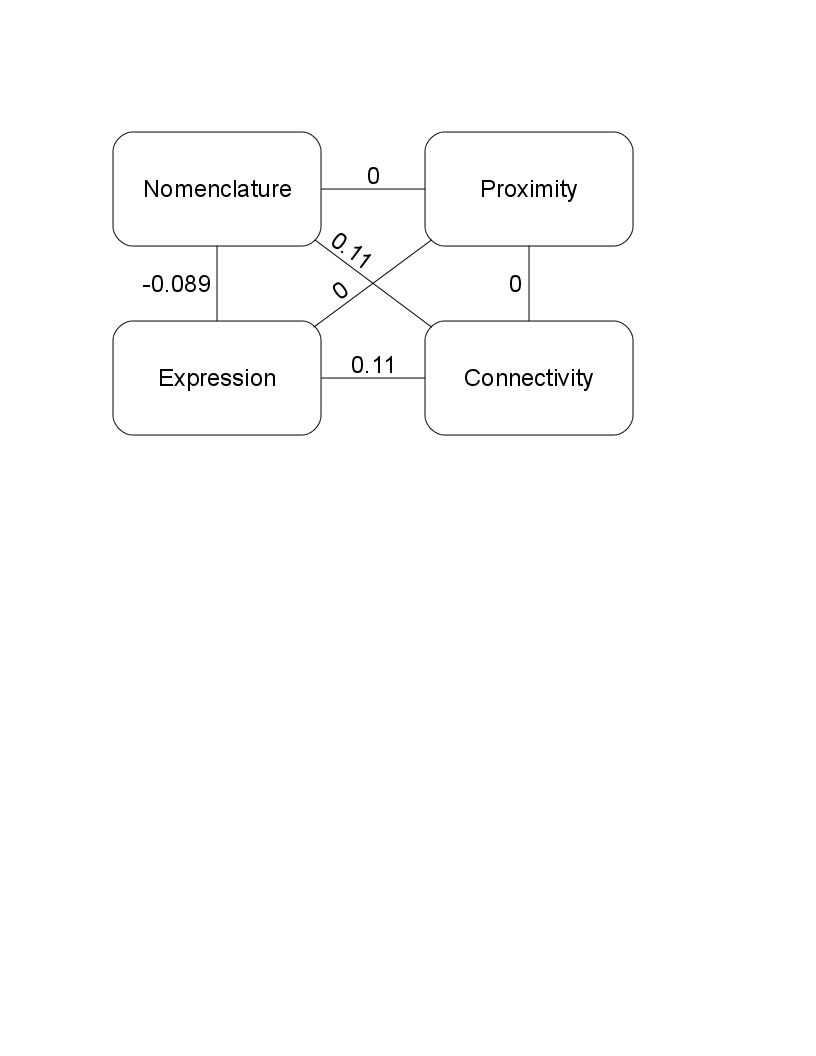

Supplement: Figure S2 — Mantel correlation between different matrices after controlling for proximity. The 141 regions with incoming connectivity information were used to generate the correlations. (0.06 MB TIF) [file pcbi.1001049.s003.tif]

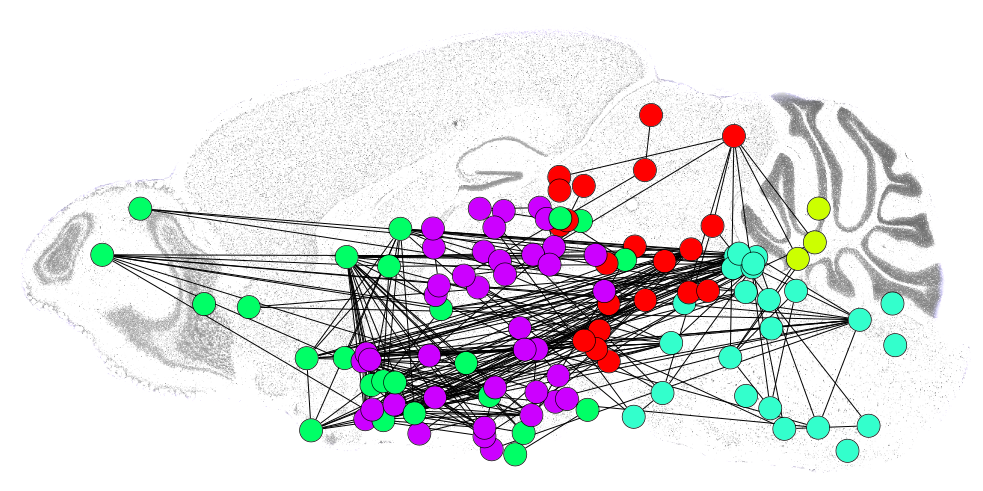

Supplement: Figure S3 — Intersection of the top 10th percentile of brain region pairings for connectivity and gene expression correlations. Using outgoing proximity controlled connectivity. With expression correlation derived from only the top outgoing genes. Colors represent five major brain divisions: cerebellum (yellow), cerebrum (green), hindbrain (blue), interbrain (purple) and midbrain (red). (0.49 MB TIF) [file pcbi.1001049.s004.tif]
